# Dynamic myocardial depression in septic cardiomyopathy

**DOI:** 10.1186/cc13439

**Published:** 2014-03-17

**Authors:** A Darawshe, D Levingston, Y Haviv

**Affiliations:** 1 The Chaim Sheba Medical Center affiliated to the Tel-Aviv University, Ramatgan, Israel

## Introduction

Depression of left ventricular contractility appears in many diseases resulting from various etiology factors. One of the most interesting features of septic cardiomyopathy is the significant dynamic myocardial depression that is commonly observed. In this context, the objective of the present work is to characterize clinically, by laboratory, by echocardiography, and by invasive measures the patients with septic cardiomyopathy.

## Methods

A single-center database investigates all patients who were admitted and treated for severe sepsis or septic shock in the ICU over a period of 2 years (November 2011 to October 2013), and who were discharged with a diagnosis of septic cardiomyopathy or new left ventricular dysfunction. The clinical, laboratory, echocardiography, and invasive measures, and clinical outcome were recorded.

## Results

From the 105 patients that were investigated, 15 patients were found with septic cardiomyopathy. Septic cardiomyopathy is more prevalent among men (60%). Patients with septic cardiomyopathy have an increased prevalence of immune compromised disease (46%), and hypertension (40%). There was a need for mechanical respiratory support for 86% of patients. The improvement in cardiac function occurred at an average of 6.9 days. E. coli is the commonest bacterial pathogen (33%). Laboratory findings show elevated liver enzyme and kidney function impairment in all patients. Thirty-three percent of patients were treated with N-acetyl cysteine, and 46% were treated with renal replacement therapy. High CRP was observed in all patients. Paroxysmal atrial fibrillation was diagnosed in 46%. Invasive measures in 50% of the patients have demonstrated high cardiac index (CI) and low systemic vascular resistant index (SVRI) on their admission, and 93% demonstrate low CI and high SVRI a few hours later. Hospitalization stay was between 3 and 42 days with an average of 14.6 days.

## Conclusion

Septic cardiomyopathy is more common among immune compromised patients. It is characterized by dynamic changes in the cardiac function based on echocardiography and invasive measures. A persistent hyperkinetic state was associated with high mortality rate. In addition to echocardiography follow-up, invasive monitoring even in their admission is of great importance for more effective and adequate treatment.

